# Distribution of 28 kDa Calbindin-Immunopositive Neurons in the Cat Spinal Cord

**DOI:** 10.3389/fnana.2015.00166

**Published:** 2016-01-28

**Authors:** Natalia Merkulyeva, Aleksandr Veshchitskii, Felix Makarov, Yury Gerasimenko, Pavel Musienko

**Affiliations:** ^1^Laboratory of Neuromorphology, Pavlov Institute of Physiology RASSaint Petersburg, Russia; ^2^Laboratory of Neuroprosthetics, Institute of Translational Biomedicine, Saint Petersburg State UniversitySaint Petersburg, Russia; ^3^Laboratory of Motor Physiology, Pavlov Institute of Physiology RASSaint Petersburg, Russia; ^4^Laboratory of Neurophysiology and Experimental Neurorehabilitation, Children’s Surgery and Orthopedic Clinic, Department of Non-pulmonary Tuberculosis, Research Institute of PhthysiopulmonologySaint Petersburg, Russia

**Keywords:** spinal cord, gray matter, interneurons, Ca^2+^-binding proteins, calbindin-28 kDa

## Abstract

The distribution of vitamin D-dependent calcium-binding protein (28 kDa calbindin) was investigated in cat lumbar and sacral spinal cord segments (L1-S3). We observed specific multi-dimensional distributions over the spinal segments for small immunopositive cells in Rexed laminae II-III and medium-to-large cells of varying morphology in lamina I and laminae V-VIII. The small neurons in laminae II-III were clustered into the columns along the dorsal horn curvature. The medium-to-large cells were grouped into four assemblages that were located in (1) the most lateral region of lamina VII at the L1-L4 level; (2) the laminae IV-V boundary at the L5-L7 level; (3) the lamina VII dorsal border at the L5-L7 level; and (4) the lamina VIII at the L5-S3 level. The data obtained suggest that the morphological and physiological heterogeneity of calbindin immunolabeling cells formed morpho-functional clusters over the gray matter. A significant portion of the lumbosacral enlargement had immunopositive neurons within all Rexed laminae, suggesting an important functional role within and among the spinal networks that control hindlimb movements.

## Introduction

Calcium ions (Ca^2+^) play an important regulatory and structural role in a wide range of biological processes (Nordin, 1988; Pochet et al., 2000; Sabatini et al., 2001; Franks and Sejnowski, 2002). As a second messenger, Ca^2+^ is involved in the regulation of intracellular functions (Samoilov, 1999). Maintaining the stable neuronal Ca^2+^ transmembrane gradient is an important aspect for growth of neuronal processes and synaptic transmission (Heizmann, 1993). Ca^2+^ signal transduction into intracellular response is provided by Ca^2+^-binding proteins. Some of these proteins, including 28 kDa calbindin, parvalbumin and calretinin, are members of the EF-hand family, which is characterized by two alpha helices linked by a short loop region that usually binds calcium ions (Grabarek, 2006; Schmidt, 2012).

Interneurons in the central nervous system (CNS) highly express specific Ca^2+^-binding proteins and can be used as a specific markers for cell functional types (Hof and Nimchinsky, 1992; Heizmann, 1993; Baizer and Baker, 2005); 28 kDa calbindin plays a major role as an intracellular Ca^2+^ buffer (Nägerl et al., 2000). Its high concentration has been observed in the cerebellar Purkinje cells, in hippocampal granule cells (Baimbridge et al., 1992; Schwaller et al., 2002), and thalamic and cortical “matrix cells” that are characterized by spreading interneuronal connections that synchronize thalamocortical elements into coherent active networks (Jones, 2001).

Neurons expressing 28 kDa calbindin have also been revealed in the spinal cord (Anelli and Heckman, 2005; Porseva et al., 2014). It has been suggested that calbindin is a specific protein of the excitatory amino acid neuron subpopulation (Antal et al., 1991). It was found that calbindin 28 kDa is predominantly distributed in lamina I and ependymal cells. Numerous calbindin-immunopositive cells are abundant in the substantia gelatinosa (Antal et al., 1991; Ren and Ruda, 1994). A group of calbindin-positive neurons belong to the Renshaw cell population in rodents, primates and cats (Arvidsson et al., 1992; Sanna et al., 1993).

Because Ca^2+^-binding proteins are expressed in neuronal populations with specific functional features, we initiated studies to determine the 3D distribution and selected features of calbindin-positive cells among the lumbosacral spinal cord segments and laminae. We hypothesize that such comprehensive neuromorphological evidence, as part of multidimentional spinal infrastructure characterization, is necessary and useful for ongoing and future morphological/functional studies of the neuronal networks participating in different types of spinal functions, such as sensorimotor activity (locomotor and postural control; Deliagina et al., 1983; Orlovsky et al., 1999; Gerasimenko et al., 2008; Musienko et al., 2010, 2014) and visceral control (bladder function; Horst et al., 2011, 2013).

## Materials and Methods

### Animals and Perfusion

All experimental procedures were approved by the Ethics Commission of the Pavlov Institute of Physiology. Experiments were performed in accordance with the requirements of Council Directive 2010/63EU of the European Parliament regarding the protection of animals used for experimental and other scientific purposes. Five normal pigmented adult cats of both sexes [four males, K7, K11 (only segment L4 was used), K12, and K15; and one female, K8] were used for this investigation. Under deep anesthesia (Isoflurane) all animals were perfused transcardially with 0.9% NaCl (2.0 L) in 0.1 M phosphate-buffered saline (PBS) at pH = 7.4, followed by 4% paraformaldehyde (2.0 L) in 0.1 M PBS at pH = 7.4. After perfusion, the spinal cord was removed from the spine and stored in 20 and 30% sucrose until it sank. The lumbosacral cord was divided into segments based upon the grouping of the dorsal rootlets. The L1-S3 segments were cut on a freezing microtome into 50 μm frontal sections, or 40 μm horizontal sections. Sections were collected in 0.1 M PBS, pH = 7.4.

### Calbindin 28 kDa Immunohistochemical Staining

Slices were processed as free floating. Between all procedures, the slices were washed 3 × 5 min in 0.01 M PBS. To unmask any antigens, sections were processed in 1% NaBH_4_ for 30 min; endogenous peroxidase activity was blocked by incubating the sections in 0.3% H_2_O_2_ for 15 min. The sections were then incubated for 1 h in 10% normal goat serum (NGS, Vector Labs) in PBS to block non-specific staining. Triton X-100 (0.3%) was added for this and subsequent incubations to enhance antibody penetration.

The sections were incubated for 70 h at room temperature in monoclonal mouse primary antibodies to 28 kDa calbindin (C9848, Sigma-Aldrich, St. Louis, MO, USA, 1:3000 dilution, 1% NGS with 0.3% Triton and 0.1% NaN_3_ were added). Then, the slices were incubated in secondary antibodies (biotinylated anti-mouse IgG, BA-9200, Vector Laboratories, Peterborough, UK, 1:400 dilution, 0.1% NaN_3_ was added) for 1 day, followed by incubation in avidin-biotin horseradish-peroxidase complex (ABC Elite system, Vector Labs) for 1 h. The peroxidase reaction was visualized with a mixture of diaminobenzidine (DAB), NiCl and 0.3% H_2_O_2_ (Vectastain DAB kit, Vector Labs). After washing in distH_2_O, sections were mounted, dehydrated, cleared and placed under coverslips.

### Antibody Characterization

The mouse monoclonal antibody against 28 kDa calbindin from bovine kidney calbindin-D was characterized by the manufacturer with an immunoblotting analysis. The manufacturer guarantees that the antibody does not react with other members of the EF-hand family such as calbindin-D-9K, calretinin, myosin light chain, parvalbumin, S-100a, S-100b, S100A2 (S100 L) and S100A6 (calcyclin). The 28 kDa calbindin antibody stained neuronal somas and processes and allowed the characterization of features of the spinal cord by calbindin labeling, such as a dark stripe corresponding to laminae II-IV, which contain small dark labeled neurons, as demonstrated in previous publications (Antal et al., 1991; Anelli and Heckman, 2005). As a control for antibody specificity, sections were processed in NGS alone. No staining was observed in this case.

### Image Acquisition and Data Analysis

**Figure 1:**
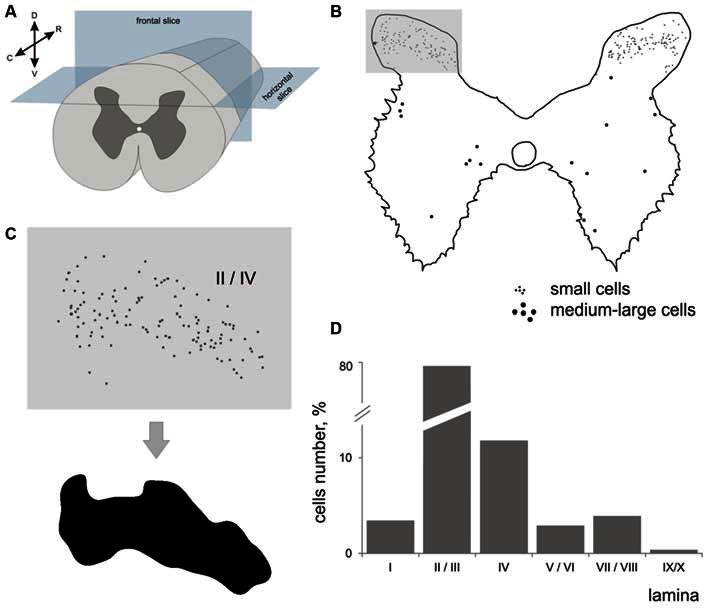
**Labeled cells analysis. (A)** Scheme for frontal and horizontal slice preparation; **(B)** Gray matter at the frontal slice; small labeled cells are marked by small dots, medium-to-large cells are marked by gross dots; shaded rectangle—the part of the dorsal horn containing laminae II-III. **(C)** A labeled area assessment in shaded rectangle at **(B)**; Upper image—an initial 2-D pattern of labeled cells in laminae II/III, low image—a blurred and contrasted 2-D pattern of labeled cells in laminae II/III. **(D)** Diagram of labeled cell distributions in different laminae (summed by all segments). *X* axis—laminae, *Y* axis—number of cells. R, C, D, V—rostral, caudal, dorsal and ventral directions.

Images were acquired with an Olympus microscope (Olympus Corporation, Tokyo, Japan, a 10× objective) using a Nikon photo camera (Nikon Corporation, Tokyo, Japan) mounted on the microscope. All images were then processed with Adobe Photoshop (Adobe Systems Inc., San Jose, CA, USA), adjusting for brightness, contrast, and sharpness to make contoured images. While counting images, small-cells were labeled with small dots, and medium-to-large cells were labeled with large dots (Figures 1A,B). For quantitative analysis for immunopositive neuron distribution in the spinal cord gray matter in the lumbar and sacral segments (L1-S3), all cells were counted separately in all laminae in all segments. For every segment, three slices were used (in caudal, medium and rostral part of segment), and the total number of cells was represented in appropriate tables and figures. Data from the right and left spinal halves were averaged because no notable differences were detected between them. The area of labeled neurons was measured with ImageJ software. The area of the small neurons in laminae II-IV was measured semi-automatically based upon the differences in cell and background brightness; the medium and large cell areas in all laminae were measured manually. In any case, we tried to measure an area only for the least damaged neurons. The average of their areas ± a 95% confidence level (CL, for normally distributed values for small cells in laminae II-IV) or ± a standard error of the mean (SEM, for abnormally distributed values for medium and large cells) were calculated. All diagrams were created in Microsoft Excel.

Because the area of segment’s gray matter in a given segment can influence the number of immunopositive cells that were visualized, we assessed the number, cell density and laminar distribution of immunopositive cells. Because the cells were dispersed in laminae II-IV non-homogeneously, we assessed a density of distribution with regard to area of a locus containing immune-positive cells. The area of these loci was calculated semi-automatically using a spatial frequency filtration algorithm (Merkulyeva and Nikitina, 2012). Briefly, the region containing labeled neurons in each image was contoured (Figure 1C) to assess the area and cell density.

## Results

The largest numbers of 28 kDa calbindin-immunopositive neurons were observed in laminae II and III of the dorsal horn (77% from total labeled cells). The amount of calbindin neurons in lamina IV was 11%; in lamina I, 3.5%; in laminae V-VI, 3%; and in laminae VII-VIII, 4%. Labeled neurons in laminae IX and X were rare (Figure 1D).

### Lamina I

**Figure 2:**
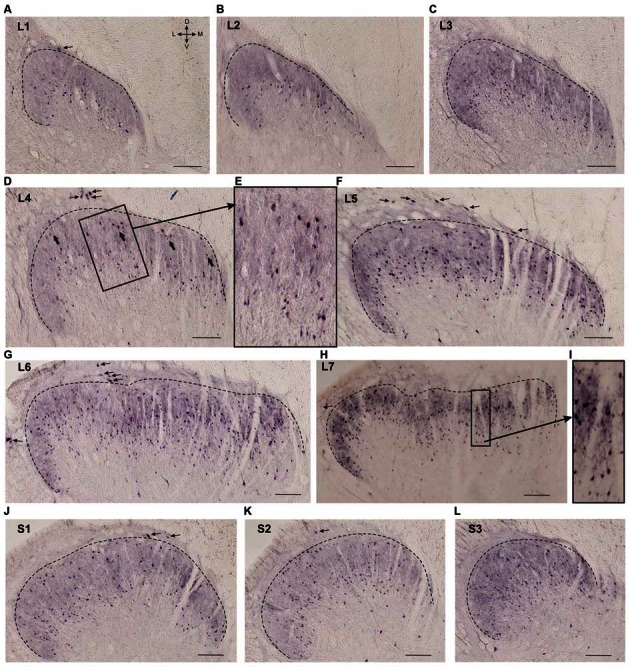
**Calbindin-immunopositive neuron distribution in different segments of the dorsal horn. (A–L)** Dorsal horn frontal slices; the laminae I-II boundary is marked by a black dashed line. **(E)** Enlarged area of labeling (in rectangle at **D**). Gross labeled cells in d are indicated by thick arrows. **(I)** Enlarged area of labeling (in rectangle at **H**). Lamina I immunopositive cells are marked by thin arrows. D, V, M and L—dorsal, ventral, medial and lateral directions. Calibration marker is 100 μm.

**Figure 3:**
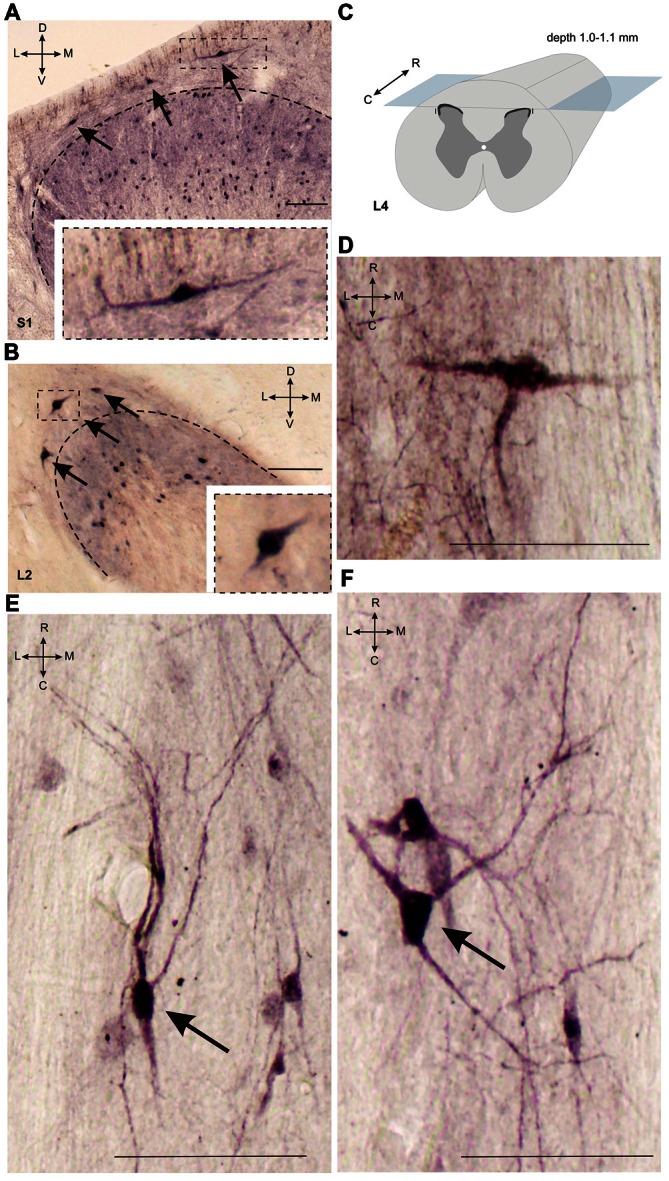
**Calbindin-immunopositive neuronal distribution in lamina I of gray matter. (A,B)** Dorsal horn frontal slices at segment S1 **(A)** and segment L2. **(B)** Labeled cells are indicated by arrows; laminae I-II boundary is marked by black dashed line. Inserts in bottom—enlarged cells (in squares). **(C)** A schematic view of horizontal slice preparations; gray—gray matter, dark gray—laminae II-III. **(D–F)** Labeled neurons at horizontal slices (depth 1.0–1.1 mm). Arrows mark neurons described in text. D, V, R, C, M and L—dorsal, ventral, rostral, caudal, medial and lateral directions. Calibration marker is 100 μm.

It was difficult to distinguish lamina I (a marginal layer) from overlying white matter. We attributed this to neurons lying above the upper boundary of the intensive laminae II-III labeling belonging to lamina I (Figure 2). Three main types of large neurons (averaged body area 280–515 μm^2^, Table 1) were visualized in lamina I. In the frontal sections, all had oval-to-triangle bodies, and only one type of cell differed from the other cells: bipolar neurons oriented parallel to the curvature of dorsal horn (Figures 3A,B). It was obvious in the horizontal slices that these bipolar cells also had a third short dendrite, which was oriented in a caudal direction (Figures 3C,D). The second and third types of immunopositive neurons could be separated only in horizontal slices; and one of them had an elongated soma and long dendrites (up to 650 μm) oriented rostro-caudally (Figure 3E). The others had plural dendrites oriented without any particular direction (Figure 3F). Scattered pale cells with small oval shaped soma, similar to cells in laminae II-III, were rarely visualized in lamina I.

**Table 1:** **Mean soma size of calbindin-positive neurons in different laminae of the spinal cord gray matter in four cats (μm^2^ ± CL or ± *SEM*, see M&M)**.

	K7	K8	K12	K15	Averaged
Lamina I	365 ± 61	384 ± 96	462 ± 62	465 ± 61	450 ± 38
	*n* = 8	*n* = 9	*n* = 37	*n* = 2	*n* = 56
Laminae II/III/IV	73 ± 2	60 ± 2	89 ± 5	73 ± 3	76 ± 2
	*n* = 1225	*n* = 963	*n* = 548	*n* = 610	*n* = 3346
Laminae V/VI	339 ± 42	344 ± 142	430 ± 84	386 ± 48	363 ± 31
	*n* = 64	*n* = 13	*n* = 44	*n* = 67	*n* = 188
Laminae VII/VIII	385 ± 97	244 ± 118	447 ± 89	425 ± 35	373 ± 25
	*n* = 39	*n* = 19	*n* = 24	*n* = 112	*n* = 194

*CL—a 95% confidence level; SEM, standard error of the mean; n, number of cells*.

**Figure 4:**
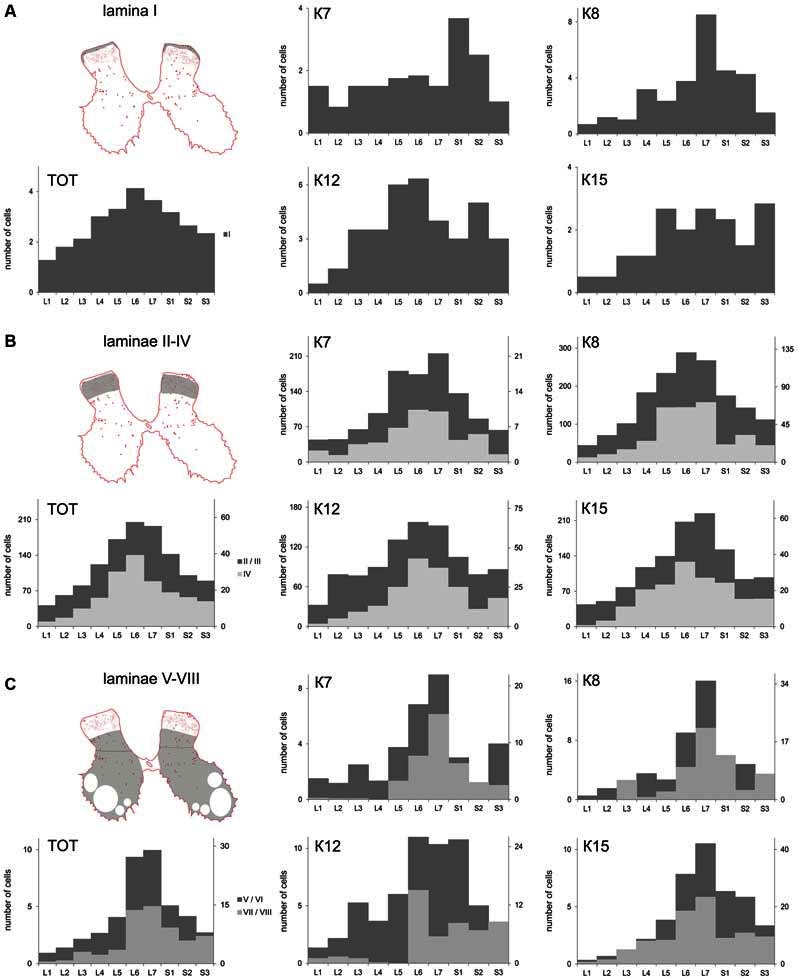
**Calbindin-labeled neuronal distribution in different Rexed laminae of the L1-S3 segments. (A)** Diagrams of the distribution of medium-to-large cells in lamina I; **(B)** Diagrams of the distribution of small cells in laminae II-IV; **(C)** Diagrams of the distribution of medium-to-large cells in laminae V-VIII. **(A–C)** left most, upper—frontal slices, regions of interest are shaded. **(A–C)**, left most, bottom—averaged from all cat data (TOT); **(A–C)** Medium and rightmost figures—diagrams for individual cats (K7, K8, K12 and K15). *X* axis—segments, *Y* axis—number of labeled cells. **(B)** Light gray—lamina IV, dark gray—laminae II-III; **(C)** Light gray—laminae V-VI, dark gray—laminae VII-VIII.

The cell count was undertaken from frontal sections, so all types of large neurons were intermingled. The maximal number of large neurons cord in all cats (K7—segments S1-S2; K8—segments L6-S2; K12—segments L5-L6 and S2; K15—segments L5-S1 and S3; Figure 4A).

### Laminae II-IV

Laminae II-III looked like a darkly stained strip and contained labeled neuronal somas and neuropil (processes oriented primarily perpendicular to the gray and white matter boundary, Figure 2I). Calbindin-immunopositive neurons were also spread throughout lamina IV. Most of the labeled cells in laminae II-IV had fusiform somas.

Neuronal distribution within laminae II-IV depended upon the spinal cord level: most of the cells in the L1-L2 segments were localized in the lower part of the labeled region (possibly in lamina III; Figures 2A,B). Starting with L3 immunopositive cells appeared in the upper part of the labeled area (Figure 2C), these neurons were larger than the underlying ones (140 ± 30 μm^2^ vs. 70 ± 35 μm^2^; Figures 2D,E). In segments L4-L5, there was a labeling pattern consisting of two stripes of cell bodies divided by an area with fewer immunopositive cells (Figures 2D–F). At L6-L7, most of the immuno-positive neurons were found throughout the laminae II-IV (Figures 2G,H). The labeling patterns in the sacral segments (Figures 2J–L) were more heterogeneous than those in the lumbar segments, and there was more variability among the cats.

**Figure 5:**
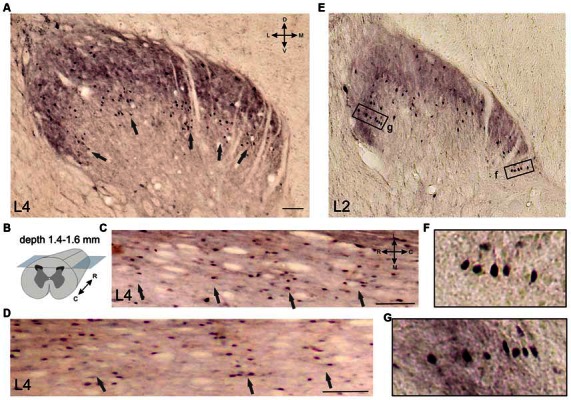
**Calbindin-immunopositive neuronal distribution in the frontal (A) and horizontal (C,D) sections of the gray matter of the dorsal horn. (A–D)** Clusters of calbindin-immunopositive cells in segment L4, laminae II-IV; clusters are indicated by arrows. **(A)** Frontal slice, **(B)** Schematic view of horizontal slice preparations, depths of 1.4, 1.5 and 1.6 mm from the pial surface. **(C,D)** Horizontal slices; clusters are indicated by arrows. **(E–G)** Columns of calbindin-immunopositive cells in laminae II-III. **(F,G)** Enlarged regions in rectangles. D, V, R, C, M and L—dorsal, ventral, rostral, caudal, medial and lateral directions. Calibration marker is 100 μm.

**Figure 6:**
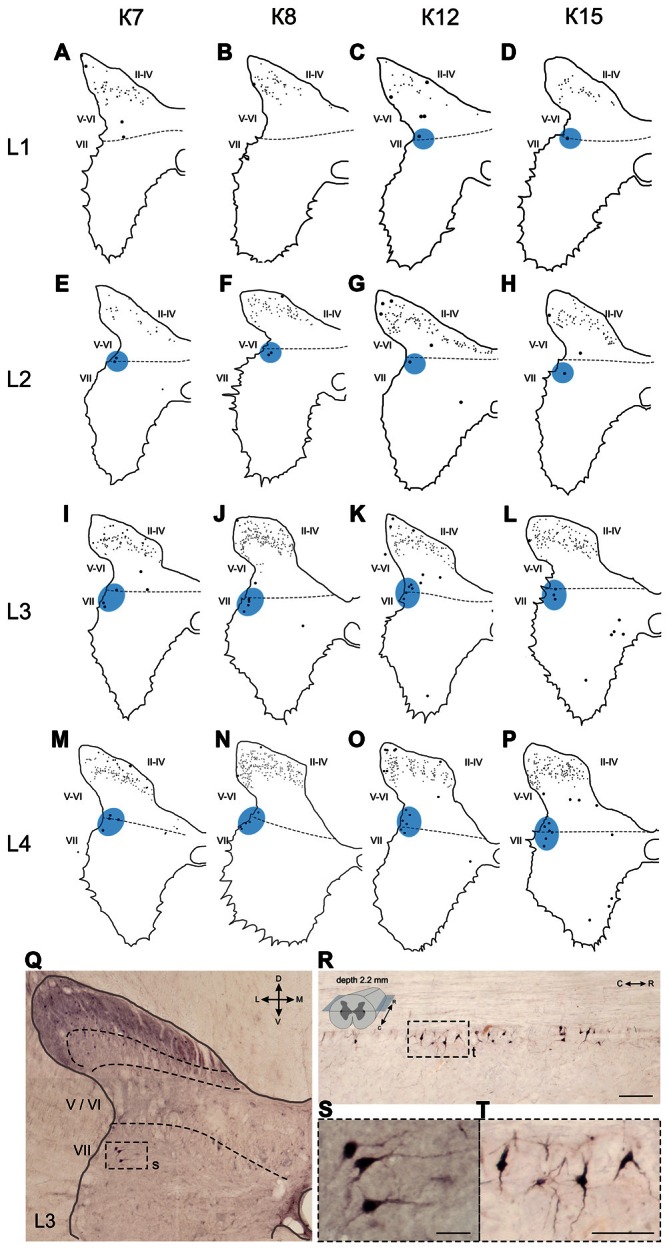
**Calbindin-immunopositive neuronal distribution in the gray matter of L1-L4 segments. (A–P)** Contoured images of frontal slices in segments L1 **(A–D)**, L2 **(E–H)**, L3 **(I–L)** and L4 **(M–P)** of cat K7 **(A,E,I,M)**, K8 **(B,F,J,N)**, K12 **(C,G,K,O)** and K15 **(D,H,L,P)**. Immunopositive neurons in laminae II-IV are marked by small dots; those in laminae V-VIII—by gross dots; laminae VI-VII boundary is marked by a dashed line. Rexed laminae are indicated by Roman numerals. Lateral groups of labeled cells are shaded in blue. **(Q–T)** Labeled cells groups in lamina VII, lateral image. **(Q,S)** Frontal slice (segment L3), **(R,T)** horizontal slice (segment L4, depth 2.2 mm, only left hemisphere is shown). The laminae III/IV, IV/V and VI-VII boundaries are marked by dashed lines. Rexed laminae are indicated by Roman numerals. D, V, M and L—dorsal, ventral, medial and lateral directions. Calibration marker is 100 μm.

Several frontal slices at the L3-L7 level contained 3–6 clusters of immunopositive cells distributed in laminae II-IV (Figures 5A, 6O). Medial neuron clusters, but not lateral clusters, were separated by afferent fibers from dorsal roots. The L4 clusters were visualized more clearly in the horizontal sections (Figures 5B–D). The clusters were distributed generally in a rostro-caudal direction. Inter-cluster distance varied from 158 to 422 μm (average 268 ± 23 μm). Therefore, the calbindin-immunopositive fusiform cells in laminae II-III were distributed non-homogenously and organized into a 3-dimensional network.

It can be seen that the immunopositive neurons occasionally organized into columnar structures; in this case, the cells were grouped in thin columns oriented perpendicular to the curvature of the dorsal horn (the white matter-gray matter boundary; Figures 5E–G).

The average number of small-labeled cells in laminae II-IV per animal was 2650 (range 2303–3204 cells). Because it is difficult to discriminate laminae II and III precisely, we combined these data. We analyzed both the quantitative and the qualitative labeling characteristics of the cells along the rostrocaudal axis. The small cell soma area was 73 ± 2 μm^2^ (*n* = 2383), with no valid rostrocaudal gradient; and we observed weak increments in cell size at the L5-L7 level in only two cats (K8 and K15; Table 2).

**Table 2:** **Number, density and soma size (μm^2^ ± CL) of 28 kDa calbindin-positive neurons in laminae II-IV of the lumbo-sacral spinal segments in four cats**.

		K7	K8	K12	K15
L1	Count	49	57	53	65
	Density, (cell/μm^2^)*1000	0.32	0.38	0.35	0.50
	Area, μm^2^	76 ± 10	52 ± 7	74 ± 17	53 ± 7
L2	Count	41	65	71	75
	Density, (cell/μm^2^)*1000	0.28	0.44	0.48	0.49
	Area, μm^2^	69 ± 6	57 ± 7	86 ± 19	69 ± 7
L3	Count	63	63	57	77
	Density, (cell/μm^2^)*1000	0.40	0.42	0.38	0.55
	Area, μm^2^	88 ± 7	60 ± 5	70 ± 8	54 ± 6
L4	Count	82	77	56	71
	Density, (cell/μm^2^)*1000	0.55	0.52	0.36	0.46
	Area, μm^2^	69 ± 5	51 ± 4	62 ± 12	67 ± 11
L5	Count	137	71	66	55
	Density, (cell/μm^2^)*1000	0.92	0.47	0.44	0.37
	Area, μm^2^	78 ± 6	45 ± 4	118 ± 16	80 ± 7
L6	Count	118	75	60	62
	Density, (cell/μm^2^)*1000	0.79	0.51	0.40	0.41
	Area, μm^2^	74 ± 3	59 ± 5	108 ± 14	84 ± 9
L7	Count	157	76	55	66
	Density, (cell/μm^2^)*1000	1.0	0.51	0.36	0.42
	Area, μm^2^	59 ± 4	65 ± 6	95 ± 13	82 ± 6
S1	Count	93	109	60	52
	Density, (cell/μm^2^)*1000	0.57	0.73	0.39	0.37
	Area, μm^2^	60 ± 6	72 ± 7	75 ± 14	88 ± 12
S2	Count	79	95	68	56
	Density, (cell/μm^2^)*1000	0.53	0.62	0.45	0.38
	Area, μm^2^	64 ± 7	54 ± 5	94 ± 10	52 ± 10
S3	Count	96	96	111	65
	Density, (cell/μm^2^)*1000	0.63	0.64	0.74	0.43
	Area, μm^2^	72 ± 13	54 ± 5	74 ± 13	71 ± 10

*CL—a 95% confidence level*.

The absolute number of labeled cells in laminae II-IV of all cats was strongly dependent on the spinal cord segment as it increased from L1 toward the lumbar enlargement, peaking at L5-L7, and then decreased in the more sacral segments (Figure 4B). Cellular density peaked at the L5-L7 level in only one animal (K7). In three other cats, only weak density increments toward to sacral region were noted (Table 2).

### Laminae V-VIII

**Figure 7:**
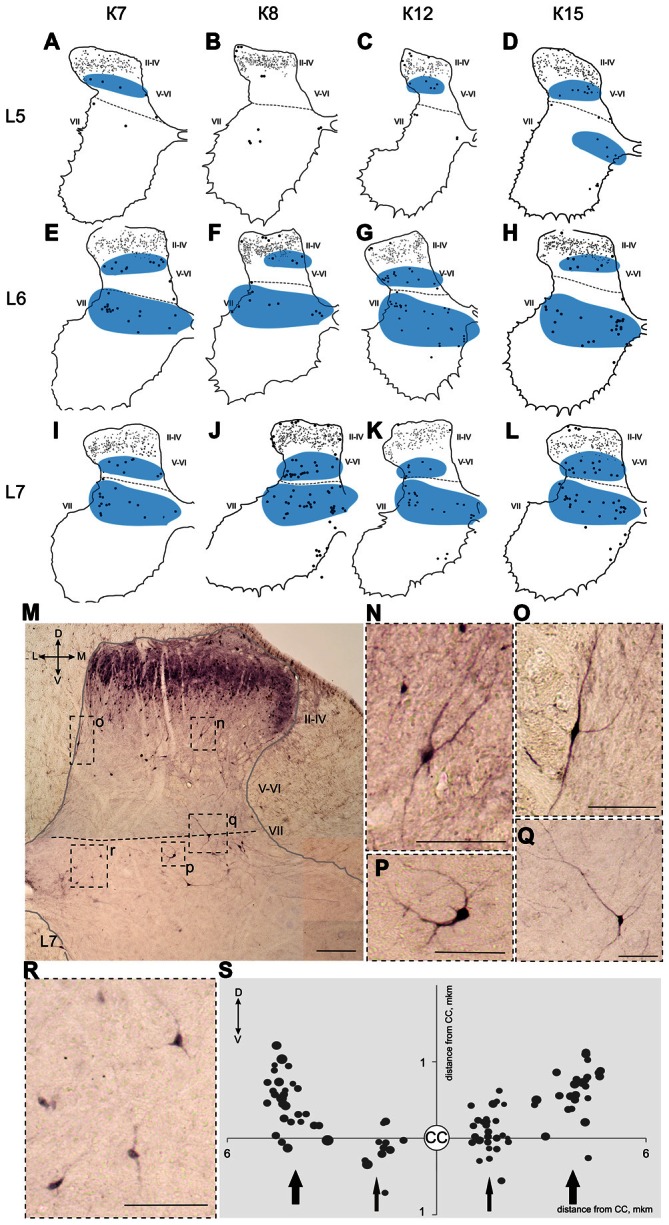
**Calbindin-immunopositive neuronal distribution in the gray matter on L5-L7 segments. (A–L)** Contoured images of frontal slices in segments L5 **(A–D)**, L6 **(E–H)** and L7 **(I–L)** of cat K7 **(A,E,I)**, K8 **(B,F,J)**, K12 **(C,G,K)** and K15 **(D,H,L)**. Immunopositive neurons in laminae II-IV are marked by small dots; those in laminae V-VIII—by gross dots; laminae VI-VII boundary is marked by a dashed line. Rexed laminae are indicated by Roman numerals. Groups of labeled cells are shaded in blue. **(M–S)** Labeled cells in laminae V-VIII. **(M)** Dorsal horn frontal slice (segment L7); **(N–R)** Enlarged cells (in dashed squares). **(S)** Bubble histogram of labeled cells located at the Laminae VI-VII boundary (as a sum of three frontal slices). Slices were centered apart from the central canal (CC, white circle). Cell area is marked by bubble size. Cell clusters are indicated by arrows (thick arrows—clusters of larger cells, thin arrows—clusters of smaller cells). D, V, M and L—dorsal, ventral, medial and lateral directions. Rexed laminae are indicated by Roman numerals. Calibration marker is 100 μm.

**Figure 8:**
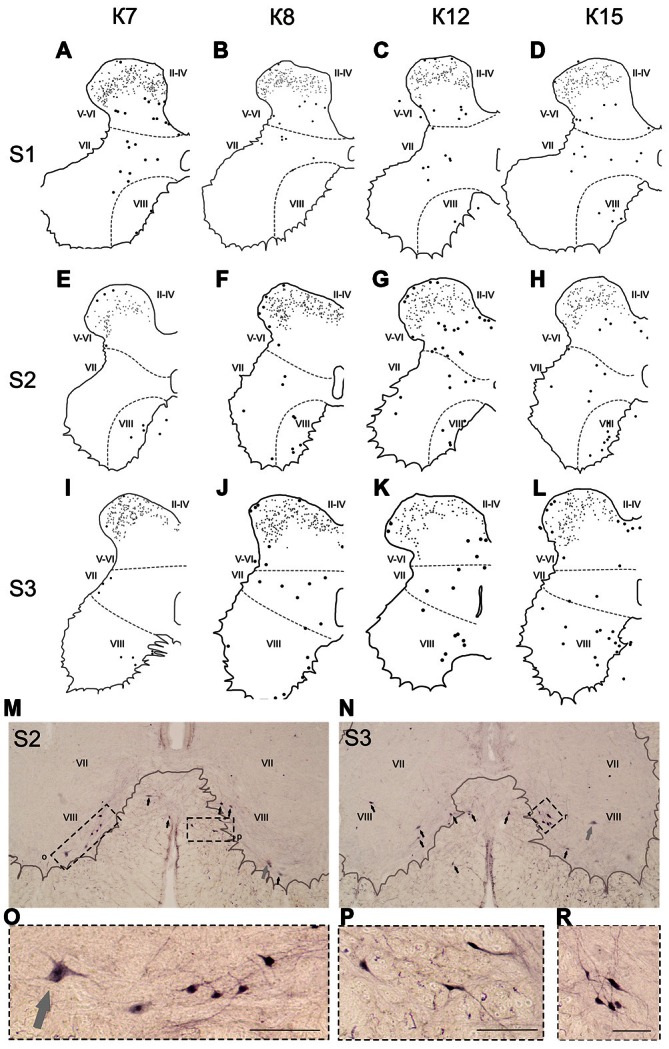
**Calbindin-immunopositive neuronal distribution in the gray matter on the S1-S3 segments. (A–L)** Contoured images of frontal slices in segments S1 **(A–D)**, S2 **(E–H)** and S3 **(I–L)** of cat K7 **(A,E,I)**, K8 **(B,F,J)**, K12 **(C,G,K)** and K15 **(D,H,L)**. Immunopositive neurons are marked by dots; laminae VI-VII and VII-VIII boundaries are marked by dashed lines. Rexed laminae are indicated by Roman numerals. **(M,N)** Frontal slices of segments S2 **(M)** and S3 **(N)**. Labeled cells are indicated by dark arrows; motor neurons are indicated by light gray arrows. **(O–R)** Enlarged cells (in dashed squares). Rexed laminae are indicated by Roman numerals. Calibration marker is 100 μm.

A range of 26–67 calbindin-immunopositive cells per animal were observed in laminae V–VI, and 38–112 immunopositive cells per animal were visualized in laminae VII-VIII. Most of the labeled cells were multipolar or pyramidal (but multipolar cells can be visualized as a pyramidal under specific tissue cutting angels); these cells were characterized by arborizing dendrites. These cells peaked at segments L5–L7 in all cats (Figures 4C, 6A–P, 7A–L, 8A–L). A single immunopositive cells were observed at the gray and white matter boundary in the medial portion of laminae V–VI; the somas and polar processes of these cells were oriented strongly along this boundary (Figures 7M,O).

Some of the labeled cells in laminae V-VIII were distributed without a clear order, but many neurons formed groups symmetrically relative to the central canal. These relatively large or small groups were divided to four regions: (1) the most lateral region of lamina VII at the L1-L4 level; (2) the laminae IV-V boundary at the L5-L7 level; (3) the dorsal border of lamina VII at the L5-L7 level; and (4) lamina VIII at the sacral level.

The first group consisted of closely-packed immunopositive cells, the number of which peaked at the L3-L4 level (Figure 6). These dark stained cells had long thick dendrites. In the frontal sections, some of them looked fusiform, but a comparison of the frontal and horizontal sections revealed that some also had multipolar morphology (Figures 6Q–T).

The second and third groups were co-localized within the same slices at the L5-L7 level (Figure 7); in one animal, these groups were visualized also in rostral S1 (Figure 8A). The second group was located at the laminae IV-V boundary and consisted of small-to-mid-sized multipolar cells (363 ± 31 μm^2^, *n* = 180; Figures 7M,N). The third group was located at the dorsal border of lamina VII and was subdivided into two clusters: a medial cluster located near the laminae VII-X boundary consisting of medium-sized cells (330 ± 36 μm^2^, *n* = 45) with weak arborized dendrites and a lateral cluster consisting of larger neurons (483 ± 60 μm^2^, *n* = 56) with long ramified dendrites (Figures 7M,P,Q,R). Figure 7S shows a reconstruction of these cell groups in one animal; we combined six slices and centered them relative to the central canal. Dot size in Figure 7S reflects relative neuronal soma size, demonstrating that the cells in the lateral cluster were indeed larger than the cells in the medial cluster (Figure 7S).

In the caudal spinal segments in lamina VIII, many labeled neurons with thin long dendrites were evident (Figure 8). These cells generally appeared at the L5-L7 (Figures 7D,H,J,K,L), and became more numerous in the more sacral segments (Figures 8E–L). These immunopositive cells had fusiform or triangle-shaped somas with a mean area of 625 μm^2^ (±46), with a range of 220–1040 μm^2^. In some tissue sections, the labeled cells were tightly grouped (Figure 8O, right side; Figure 8R) but some labeled cells were observed outside of the gray matter.

### Laminae IX and X

Only scattered pale immunopositive neurons were observed in laminae IX-X (3–5 per animal), so no statistical data are available. The immunopositive cells in the most ventral portion of the gray matter (possibly lamina IX) had a large soma size (1100–3200 μm^2^) and were thus most likely motor neurons (Figures 8M–O, gray arrows).

## Discussion

Neuronal control of movement is organized by heterogeneous population of interneurons. Interneurons expressing specific Ca^2+^-binding proteins have different discharge patterns (Hof and Nimchinsky, 1992; Heizmann, 1993; Kawaguchi and Kubota, 1993; Markram et al., 2004; Baizer and Baker, 2005), so immunolabeling can be used as a functional cell type marker. In the present work, we present a detailed description of calbindin-immunopositive neurons that are distributed among different Rexed laminae over the lumbar and sacral segments of the cat spinal cord.

### Lamina I

It is known that special types of neurons are located in lamina I (Watson et al., 2008): (1) fusiform cells responding to nociception and expressing GABA and dynorphin; (2) pyramidal cells responding to thermal sensation and expressing encephalin; (3) multipolar cells responding to polymodal stimulation and expressing GABA; (4) flatted cells expressing substance P and dynorphin; and (5) T-shaped cells (Lima and Coimbra, 1986; Prescott and Koninck, 2002).

We found three main types of calbindin-immunoreactive cells in lamina I: elongated cells with principal dendrites oriented rostro-caudally, oval-shaped curved cells with dendrites primarily oriented in medio-lateral direction, and cells with dendrites extended without any particular direction. The first type possibly corresponds to fusiform cells, the second one, to pyramidal cells, and the third one, to multipolar cells (Lima and Coimbra, 1986; Watson et al., 2008). Lamina I neurons play a crucial role in the conduction of nociceptive and temperature signals to supraspinal structures (Craig et al., 2002; Spike et al., 2003). It was shown by Craig et al. (1994, 2002) that in primates, 28 kDa calbindin is labeled in lamina I cells that send spinothalamic axons to the VMpo nucleus (posterior part of thalamic ventromedial nucleus) in response to the perception of temperature and pain. They also showed an absence of any correspondence between projecting neurons and particular morphological cell types in lamina I.

The staining of 28 kDa calbindin in lamina I cells was previously shown in both rat and cat spinal cord (Yoshida et al., 1990; Gamboa-Esteves et al., 2001; Anelli and Heckman, 2005). Such neurons have been assumed to be fusiform, flattened and pyramidal in rats (Gamboa-Esteves et al., 2001), or multipolar, in cats (Anelli and Heckman, 2005). As demonstrated previously in cats, lamina I large fusiform cells were calretinin-labeled but were not calbindin immunopositive (Anelli and Heckman, 2005). Overall, 39.4% of the fusiform cells in lamina I were calbindin-positive in the rat spinal cord (Gamboa-Esteves et al., 2001). Our study confirms that the lamina I large spindle-soma cells can be calbindin-positive. We also revealed an increasing of lamina I cells in the lumbosacral enlargement.

### Laminae II-III

Laminae II-III interneurons are responsible for the transmission and modulation of somatosensory information. The neuronal organization of these laminae is heterogeneous and includes functional groups that were determined with electrophysiological and neuromorphological data (Yasaka et al., 2010). Four main neuronal types are found in laminae II-III: islet cells and central cells with dendrites expanded in the rostrocaudal direction and radial and vertical cells with dendrites expanded in the dorsoventral direction (Grudt and Perl, 2002). Accordingly, most of the calbindin-immunopositive neurons labeled in our study had dendrites oriented dorsoventrally, which presumably was indicative of radial or vertical cells.

In both cats and in rats, the spinal neurons in lamina III are larger than the corresponding cells in lamina II (Rexed, 1954; Scheibel and Scheibel, 1968; Anelli and Heckman, 2005; Watson et al., 2008). Our data, however, show that the immunopositive cells in lamina III were equal to or even smaller in size than the ones in lamina II (Figures 2D–F). In cats, the sizes of the calbindin-positive neurons in laminae II-III were comparable across different rostrocaudal levels.

The present work demonstrates that the laminae II-III immunopositive cells occurred in clusters, predominantly in the L3-L5 segments. As shown by Scheibel and Scheibel (1968) using Golgi technique, neurons in laminae II-III tended to be arranged in a columnar fashion in frontal sections, although they were thinner than the clusters observed in the present study. It was shown by Rivero-Melián and Grant (1990) and Takahashi et al. (2007) that the central projections from the lumbar dorsal root ganglia formed patches in laminae II-III in the mediolateral and rostrocaudal directions. The location, size and numbers of these patches suggest that the calbindin-clusters observed in laminae II-III may correspond to hindlimb dermatomes and dorsal horn cutaneous somatotopic organization. In this case, one functional role of these cells may be multisensory integration.

### Laminae IV-VIII

The present study confirms that heterogeneous populations of calbindin-labeled neurons are distributed in laminae IV-VIII (Anelli and Heckman, 2005; Porseva et al., 2014). The first group of calbindin-immunopositive cells was visualized in the most caudal portion of the intermediate gray matter (dorsal part of lamina VII) in segments L1-L4, which were especially clear in the horizontal slices (Figure 6R). The localization of this group and its disappearance near the L5 segment suggest that it possibly belongs to the nucleus intermediolateral (IML), which contains preganglionic sympathetic neurons. A gradient of calbindin-containing sympathetic cells in the rat IML has been reported, with most of the cells in the rostral and caudal portions of the IML and fewer cells in the central portion (Grkovic and Anderson, 1997).

Among the calbindin D28k-expressing neurons in the ventral-most zone of lamina VII, a functionally discrete subtype also expresses gephyrin (Sanna et al., 1993). These small cells (10–20 μm^2^) are likely Renshaw interneurons, which are responsible for recurrent inhibition of motor neurons (Renshaw, 1946). In contrast to rats, only 47% of the neurons classified as Renshaw cells in cats also have calbindin D28k-immunoreactivity (Carr et al., 1998). Carr et al. (1998) reported that there were approximately 750 cells per ventral horn present in the L6 segment of the cat. This may explain why only scattered small immunopositive cells were seen in the present study along the ventral margin of lamina VII. These cells, however, cannot be confidently classified as Renshaw cells based only on their morphology.

Two elongated groups of labeled cells, which were symmetrically placed relative to the central canal, were revealed at the laminae IV-V and VI-VII boundaries in the L5-L7 segments. These groups were located in similar regions, in which fibers from the alpha-ventral portion of the gigantocellular reticular formation arborize in the mouse spinal cord (Liang et al., 2015). It is important that calbindin labeling have been observed in the medial portion of this nucleus (Celio, 1990). A two-stripe pattern (in lamina V and the dorsal portion of lamina VII) was also evident for both the lateral corticospinal fiber system and the primary afferent collaterals system (Scheibel and Scheibel, 1966).

The group of labeled cells in lamina VIII of the sacral segment could be related to the spinal *nucleus of the bulbocavernosus*, which is more prominent in males than in females (Sakamoto, 2014). This was the case in our study, based on data from only one female and four male cats (Figure 8K). One other structure possibly corresponding to this group of cells is the *nucleus comissuralis. N.comissuralis* is also located in ventral horn, and has maximal expression in the second and third sacral segments (Rexed, 1954). In the caudal lumbar segments, two main subpopulations of commissural interneurons were found, those with monosynaptic input from reticulospinal, vestibulospinal neurons and group I afferents; and those with monosynaptic input from group II muscle afferents (Jankowska et al., 2005a,b,c). These cells may be linked to or part of the locomotor networks (Kiehn, 2006; Jankowska, 2008).

In general, it is important to note that total amount of labeled cells peaks in the lumbar enlargement and that a particular pattern appears in this region. Because there is evidence that calbindin is a specific marker of thalamic and cortical interneurons that synchronize specific and nonspecific elements of the thalamocortical network into coherent activity (Jones, 2001) and can also modulate activity levels within all cortical layers (Rausell et al., 1992), it can be hypothesized that similar neuronal networks exist in the spinal cord. We can suppose their distribution in the spinal areas that are involved in (1) multisensory integration (in laminae II-IV); (2) connections with particular supraspinal structures (e.g., reticular formation); or (3) commissural connections. According to our data, the candidates for functionally similar neurons can be medium-to-large calbindin-positive cells revealed at the laminae IV-V and VI-VII boundaries and in lamina VIII, as well as small cells in laminae II-IV. That a significant portion of the immunopositive neurons are located in the lumbosacral enlargement (segments L5-L7) in various Rexed laminae suggests their important functional role within and among the spinal networks that control hindlimb movements.

As for laminae II-IV, regular clusters of calbindin-positive neurons are of particular interest to researchers. It is well known that one of main principles for some brain structures is modular organization (Krasnoshchekova, 2007). Neurons inside of modules are grouped according to their responses, molecular features and connecting properties; visual cortex modules are an example of this (Hubel and Wiesel, 1977; Hübener et al., 1997; Kaas, 2012). In the cortex, we can see modules of several orders, and the highest order modules allow the processing of complex information regarding small portions of the visual space. These modules contain 1–2 calbindin clusters. Modules in any part of brain are based initially upon thin dendritic and axonal bundles. In the spinal cord, thin dendritic and axonal bundles were discovered by Ramón y Cajal (1909), and connection clusterization has also been demonstrated (Rivero-Melián and Grant, 1990; Takahashi et al., 2007), but molecular clusterization has not been performed. Therefore, we demonstrated the molecular clusterization of laminae II-IV of the dorsal horns. Features of calbindin neurons allow us to hypothesize that these clusters can be part of spinal sensorimotor and somatovisceral integration networks.

Calbindin is one possible method of characterizing the spinal neuronal infrastructure. The current paper is specifically devoted to describing the detailed distribution of calbindin 28 kDa-immunopositive neurons, their morphology and their 3D distribution in the lumbar and sacral segments of the cat spinal cord. There are other neuromorphological approaches that may be used to accomplish the above objectives. We believe that such comprehensive descriptive neuromorphological analysis is necessary and high useful as a framework for ongoing and future morphological/functional studies of the spinal neuronal networks participating in different types of sensorimotor activity (locomotor and postural function), as well as spinal neurocontrol of visceral systems (bladder function).

## Author Contributions

All authors had full access to all the data in the study and take responsibility for the integrity of the data and the accuracy of the data analysis. Study concept and design: NM, PM. Acquisition of data: NM. Analysis and interpretation of data: NM, AV. Drafting of the manuscript: NM, PM. Critical revision of the manuscript for important intellectual content: NM, PM, YG, FM. Statistical analysis: NM. Obtained funding: NM, AV. Administrative, technical, and material support: NM, AV, PM. Study supervision: NM, PM.

## Conflict of Interest Statement

The authors declare that the research was conducted in the absence of any commercial or financial relationships that could be construed as a potential conflict of interest.
