# Corrigendum: Distribution of 28 kDa Calbindin-Immunopositive Neurons in the Cat Spinal Cord

**DOI:** 10.3389/fnana.2016.00126

**Published:** 2016-12-22

**Authors:** Natalia Merkulyeva, Aleksandr Veshchitskii, Felix Makarov, Yury Gerasimenko, Pavel Musienko

**Affiliations:** ^1^Laboratory of Neuromorphology, Pavlov Institute of Physiology RASSaint-Petersburg, Russia; ^2^Laboratory of Neuroprosthetics, Institute of Translational Biomedicine, Saint Petersburg State UniversitySaint-Petersburg, Russia; ^3^Laboratory of Motor Physiology, Pavlov Institute of Physiology RASSaint-Petersburg, Russia; ^4^Laboratory of Neurophysiology and Experimental Neurorehabilitation, Children's Surgery and Orthopedic Clinic, Department of Non-pulmonary Tuberculosis, Research Institute of PhthysiopulmonologySaint-Petersburg, Russia

**Keywords:** spinal cord, gray matter, interneurons, Ca^2+^-binding proteins, calbindin-28 kDa

There was a mistake in the affiliation of the authors. Instead of “State University, Saint Petersburg, Russia” it should be “Saint Petersburg State University, Saint Petersburg, Russia”:

^*1*^*Laboratory of Neuromorphology, Pavlov Institute of Physiology RAS, Saint Petersburg, Russia*

^*2*^*Laboratory of Neuroprosthetics, Institute of Translational Biomedicine, Saint Petersburg State University, Saint Petersburg, Russia*

^*3*^*Laboratory of Motor Physiology, Pavlov Institute of Physiology RAS, Saint Petersburg, Russia*

^*4*^*Laboratory of Neurophysiology and Experimental Neurorehabilitation, Children's Surgery and Orthopedic Clinic, Department of Non-pulmonary Tuberculosis, Research Institute of Phthysiopulmonology, Saint Petersburg, Russia*

This error does not change the scientific conclusions of the article in any way.

The original article has been updated.

## Conflict of interest statement

The authors declare that the research was conducted in the absence of any commercial or financial relationships that could be construed as a potential conflict of interest.

